# Conversion of a sigma rectum pouch to a continent cutaneous urinary diversion – enhancing quality of life through a bowel-sparing surgical approach

**DOI:** 10.1007/s00423-026-04110-6

**Published:** 2026-07-08

**Authors:** Margarete Teresa Walach, Raimund Stein, Malin Nientiedt

**Affiliations:** 1 Department of Urology and Urologic Surgery, University Medical Centre Mannheim (UMM), University of Heidelberg, Theodor-Kutzer-Ufer 1-3, Mannheim, 68167 Germany; 2 Center for Pediatric, Adolescent and Reconstructive Urology, University Medical Centre Mannheim (UMM), University of Heidelberg, Theodor-Kutzer-Ufer 1-3, Mannheim, 68167 Germany

**Keywords:** Reconstructive urology, Urinary diversion, Ureterosigmoidostomy, Mitrofanoff Stoma, Bladder exstrophy

## Abstract

**Purpose:**

The sigmoid rectum pouch (SRP) is a continent anal urinary diversion that may cause long-term complications requiring revision. This study describes our bowel-sparing surgical (BSS) technique for conversion to a continent cutaneous urinary diversion (CCUD) and evaluates functional outcomes and quality of life (QOL).

**Methods:**

We retrospectively analysed all patients undergoing SRP-to-CCUD conversion at our tertiary referral center between 2015 and 2020. Clinical records and follow-up data were reviewed. Endpoints included serum creatinine, urinary tract infections, pouch capacity, urinary continence (International Consultation on Incontinence Questionnaire [ICIQ]), fecal continence (Wexner score), and QOL (EuroQol-5D-5 L). Descriptive statistics were applied.

**Results:**

Seven patients underwent conversion at a mean age of 32 years with a mean follow-up of 91 months. All procedures were successfully completed using a BSS approach preserving bowel length. No severe postoperative complications (≥ CDC 3) occurred. Renal function remained stable (mean preoperative serum creatinine 0.92 vs. 1.00 mg/dL postoperatively). Severe urinary tract infections decreased postoperatively. One-third of patients achieved full urinary continence, one-third mild, and one-third moderate incontinence. Fecal continence remained stable (four patients with perfect, three with good continence). QOL was favorable (mean EQ-5D-5 L index 0.93; mean VAS 76), with all patients reporting subjective improvement.

**Conclusion:**

BSS conversion of SRP to CCUD is a feasible option in selected patients, preserving bowel length while maintaining renal function, acceptable continence, and good QOL.

## Introduction

The Sigma Rectum Pouch (SRP/Mainz Pouch II) is a continent urinary diversion technique that creates a low-pressure reservoir from the rectosigmoid colon, designed to improve continence and protect the upper urinary tract [1]. This modification of traditional ureterosigmoidostomy was developed to overcome major complications associated with the original technique, particularly high rates of urinary incontinence, deterioration of the upper urinary tract, and recurrent pyelonephritis [2].

SRP is primarily indicated for patients requiring urinary diversion following bladder loss or severe functional impairment, including radical cystectomy for bladder cancer, bladder exstrophy, incontinent epispadias, or severe urethral trauma [3, 4]. Despite its functional advantages, the procedure may be associated with long-term complications that occasionally necessitate surgical revision. Metabolic disturbances, particularly hyperchloremic metabolic acidosis and hypokalemia, represent the most frequent adverse outcomes and may contribute to progressive renal dysfunction if inadequately managed [5, 6]. Other reported complications include upper urinary tract obstruction, recurrent urinary tract infections, pyelonephritis, progressive hydronephrosis, and, rarely, mechanical bowel obstruction [3, 7].

In selected patients, these complications may require conversion of the SRP to a continent cutaneous urinary diversion (CCUD). Indications for conversion include refractory pouchitis, recurrent urinary tract infections, persistent urinary incontinence, deterioration of anal sphincter function, or overall unsatisfactory functional outcomes. In addition, the potential risk of malignant transformation at the ureterointestinal anastomosis necessitates lifelong surveillance and may represent a further indication for conversion in specific cases [8–10].

Compared with conversion to an incontinent diversion such as an ileal conduit, CCUD offers the advantage of preserving continence and maintaining QOL, making it an attractive alternative in patients with failed SRP [11, 12]. However, this conversion typically requires additional bowel resection and should therefore adhere to the principles of bowel-sparing surgery (BSS) to preserve intestinal length and function as much as possible.

Due to the rarity of these conditions, converting from SRP to CCUD is a highly specialised procedure. Therefore, the aim of this study was to describe our center’s BSS approach for converting an SRP to a CCUD and to evaluate postoperative clinical outcomes and patient-reported QOL.

## Materials and methods

### Study design, study population and data collection

A retrospective analysis was performed of all patients undergoing conversion from continent anal urinary diversion (SRP) to CCUD at our center between October 2015 and March 2020. Clinical records, including inpatient and follow-up visits, were reviewed.

Urinary tract infections were documented by microbiological culture. Renal function was assessed using serum creatinine and eGFR. Postoperative complications were graded according to the Clavien-Dindo classification (CDC) within 30 days. Urinary continence was evaluated using the ICIQ-UI-SF and fecal continence using the Wexner score [13–15]. Pouch volume was patient-reported. QOL was assessed using the EQ-5D-5 L and the visual analogue scale (VAS) [16].

The study was conducted in accordance with the Declaration of Helsinki and approved by the University of Heidelberg Ethics Committee II (reference number 2021 − 893).

### Outcomes

**Fig. 1:**
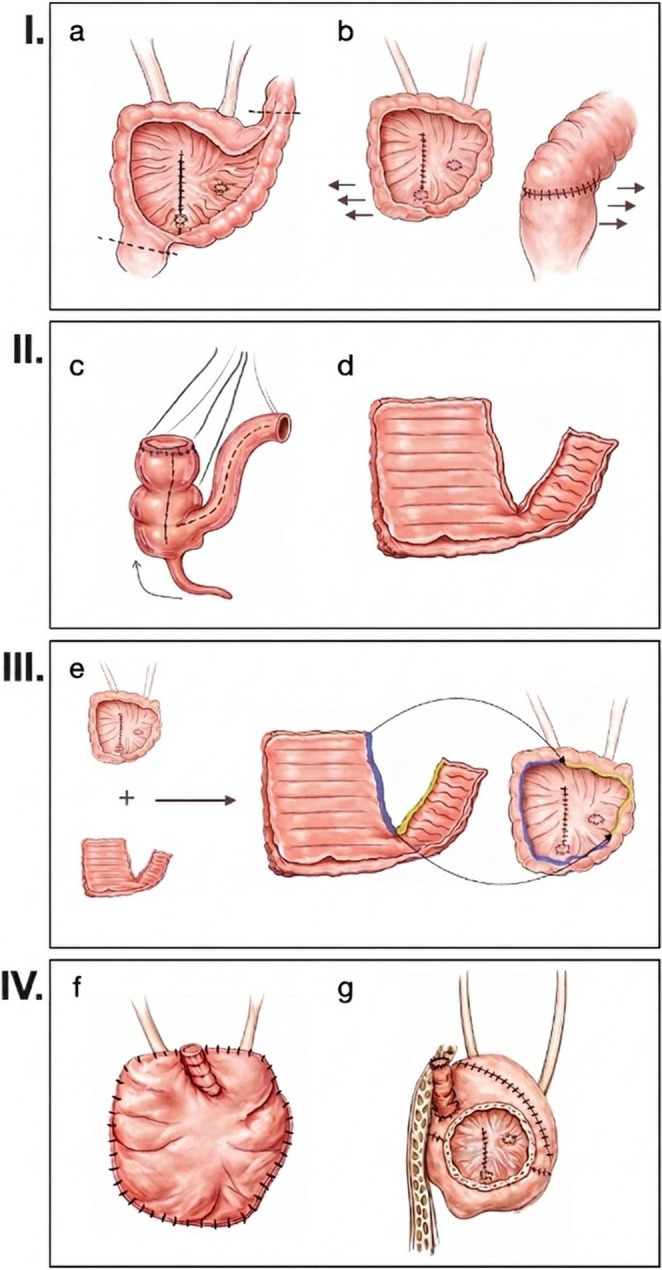
Step-by-step illustration of the applied surgical technique **I.a** Mobilisation and resection of the SRP, **I.b** Lateralisation of anastomosed proximal sigmoid colon and rectum and medialisation of resected pouch, **II.c** Mobilisation of the ileocecal segment and bowel preparation, **II.d** Detubularization of the colon and ileal segments and embedding of the appendix into the taenia libera, **III.e** Reconstruction of the continent urinary reservoir using the ileocolonic pouch and the preserved rectosigmoid pouch, **IV.f** Exteriorisation of the appendix through the abdominal wall and fixation to fascia and skin to create an umbilical stoma, **IV.g** Side view of the completed pouch fixed to the lateral abdominal wall

The primary endpoint of this study was the assessment of functional outcomes in the patient cohort based on clinical and laboratory parameters as well as the evaluation of QOL. Furthermore, our aim was to provide a detailed, step-by-step description of our surgical approach during the conversion procedure, illustrated by surgical drawings (Fig. 1).

### Statistical analyses

Descriptive statistics included frequencies and proportions for categorical variables. For continuously coded variables, means, medians, and ranges were reported. No inferential statistics were performed due to small sample size. All statistical analyses were carried out with JMP^®^ v16 (SAS Institute, Cary, NC, USA).

## Results

### Surgical technique: bowel-sparing conversion of SRP to CCUD

#### Step I – Mobilisation and resection of the SRP/ureterosigmoidostomy

Access is obtained via the previous midline laparotomy. After adhesiolysis, the sigmoid colon, rectum, and ureters are mobilised. The ureters are dissected to the ureterosigmoid junction and secured.

The rectosigmoid pouch is mobilised for partial resection while maximally preserving bowel length in accordance with BSS principles. Continuity is restored by a tension-free sigmoid–rectal anastomosis (Fig. 1 Ia–b).

Depending on ureteral conditions, three approaches are used:

1. Direct ureteral preservation: resection of the pouch including ureteric implantation sites, followed by re-anastomosis.
2. Ureter excision with mucosal cuff: ureters are excised with a small intestinal cuff for frozen section, defects are closed, and the rectosigmoid pouch plate is preserved for augmentation.
3. Classic ureterosigmoidostomy: ureters are excised at the rectal entry, defects closed, and, if required, the sigmoid segment is preserved as a pouch plate.

In all cases, bowel preservation is prioritised, with maximal retention of the rectosigmoid segment for subsequent augmentation. When ureters are excised (options 2 and 3), they are reimplanted into the newly constructed reservoir. The preserved pouch plate is medialised for augmentation (Fig. 1 Ib).

#### Step 2: Mobilisation of the ileocecal segment and bowel preparation

The ileocecal segment and, if required, the ascending colon are mobilised according to the augmentation needs.

Bowel resection is performed, using only the minimal intestinal length required for augmentation. Typically, this includes 10–15 cm of the cecum or ascending colon and 12–25 cm of terminal ileum. See Fig. 1 IIc.

Following resection, intestinal continuity is restored by a single-layer ileo-ascending anastomosis using a continuous seromuscular suture. The appendix is prepared as a potential continence channel by resecting the tip and dilating the lumen to 14–16 Ch. The colon and ileal segments are detubularised along the taenia libera to create a U-shaped pouch plate. If required, the appendix is embedded seromuscularly into the taenia libera of the colon segment to prepare the continence mechanism. See Fig. 1 IId.

#### Step 3 – Reconstruction of the continent urinary reservoir

Pouch configuration:

The U-shaped ileocolonic pouch is anastomosed to the preserved rectosigmoid segment to create a low-pressure, high-capacity reservoir (Fig. 1 IIIe). Preserved ureters remain in situ.

Ureter implantation:

When required, ureters are reimplanted into the terminal ileum, utilising the ileocecal valve for anti-reflux protection. Implantation is performed according to Nesbit, Wallace, or Aboul-Enein techniques.

For narrow ureters, submucosal tunnelling (2–2.5 cm) is applied. Ureters are splinted with 6–8 Ch catheters and secured with absorbable sutures.

#### Step 4 – Construction of the continent stoma

The continence mechanism is typically created using the appendix as a catheterisable channel (Mitrofanoff principle), brought out as an umbilical stoma (Fig. 1 IVf–g). Umbilical reconstruction may be required in patients with bladder exstrophy.

If the appendix is unavailable, alternatives include a Monti tube, tapered ileum, or ileal nipple. The channel is fixed within the umbilical funnel and a 10–16 Ch catheter is inserted.

#### Step 5 – Finalisation

The reservoir is tested for leakage (200–300 mL saline) and fixed to the abdominal wall. Ureteral stents are exteriorised.

A drain may be placed. The procedure is completed with retroperitonealisation and layered closure.

### Cohort

Seven patients were included. The underlying conditions included bladder exstrophy in four patients, incontinent epispadias in two patients and s.p. resection of the urethra after urethral prolapse in one patient. Six of the patients had initially undergone treatment with a classical SRP, while one patient had undergone ureterosigmoidostomy.

Prior to converting the anal urinary diversion to a continent cutaneous urinary diversion, five of the seven patients underwent additional surgical procedures related to their primary condition. These included ureteral reimplantation, stone removal procedures, genital reconstruction, vasectomy, incisional hernia repair, appendectomy and rectal prolapse resection.

Indications for BSS surgery tended to be multifactorial. The most common indication was recurrent pyelonephritis (*n* = 5). Other indications included impaired urinary transport (*n* = 2), accompanied by urolithiasis and renal function deterioration, as well as anal incontinence (*n* = 2).

Following conversion, four patients required additional surgical interventions during follow-up. These included stomal revision in four patients, open pouch stone removal in two patients and, in individual cases, lymphocele resection and ureteral reimplantation. Patient characteristics are summarised in Table 1.

**Table 1:** Patient characteristics

Patient	Age (years)	Indication	Procedures between SRP and CCUD (*n*)	Procedures after CCUD (*n*)	Follow-up (months)
1	28	– Recurrent UTI – Unilateral PRR – Bilateral UTD	None	None	121
2	30	– Unilateral PRR – Urolithiasis	– Bilateral ureteral reimplantation – 2 x PCNL	– Stoma revision – Pouch stone removal	100
3	28	– Recurrent UTI – Unilateral UTD	None	– 2 x Stoma revision	69
4	34	– Recurrent UTI – Bilateral UTD	– Unilateral ureteral reimplantation	– Stoma revision – Lymphocele resection – Continence mechanism revision	90
5	21	– Recurrent UTI – Anal incontinence – Bilateral UTD – Urolithiasis	– Genital reconstruction – Vasectomy – 3 x URS – 3x PCNL	None	68
6	41	– Recurrent UTI	– Unilateral ureteral reimplantation	None	108
7	41	– Anal incontinence	– Genital reconstruction – Appendectomy – Incisional hernia repair – Rectal prolapse resection	– Unilateral ureteral reimplantation – Continence mechanism revision – Pouch stone removal	82

SRP = sigmoid rectum pouch, CCUD = continent cutaneous urinary diversion, UTI = urinary tract infection, PRR = pouch-related reflux, UTD = urinary transport disorder, PCNL = percutaneous nephrolithotomy, URS = ureteroscopy

At the time of surgery, the mean patient age was 32 years (range 21–41 years). The mean body mass index (BMI) was 25.0 kg/m² (SD 4.76). All patients had an ASA score of II. Preoperative serum creatinine levels were normal in all patients, with a mean value of 0.92 mg/dL (SD 0.18).

### Perioperative outcomes

The mean operative time was 695 min (SD 57), and the mean estimated blood loss was 429 mL (SD 170). No patient underwent concomitant cystectomy.

Postoperatively, one-third of the patients experienced no complications. Importantly, no postoperative complications of Clavien–Dindo classification grade ≥ 3 were observed in the other cases. Ureteral stents were removed between postoperative days 10 and 17. No cases of acute renal failure occurred in the immediate postoperative period.

The mean length of hospital stay was 15 days (SD 3). Prior to removal of the stoma catheter, four patients underwent pouchography. Clean intermittent self-catheterization (CISC) was initiated between three and five weeks postoperatively. Postoperative vitamin B12 levels were regularly assessed in all but one patient and remained within the normal range in all patients with available follow-up data. Histopathological examination of all specimens revealed no evidence of malignancy.

### Functional outcomes and follow-up

The mean follow-up duration was 91 months (SD 20). At the latest follow-up, renal function remained stable for all patients, with serum creatinine levels within the normal range (mean 1.00 mg/dL, SD 0.14) and an estimated mean glomerular filtration rate (eGFR) of 79 ml/min (SD 16.21).

The mean pouch capacity at the final follow-up was 670 ml (SD 180). Regarding urinary continence, two patients reported complete continence (ICIQ-SF = 0), three reported mild incontinence (ICIQ-SF 1–5) and two reported moderate incontinence (ICIQ-SF 6–12).

Bowel continence outcomes were favourable. Four patients reported perfect continence (Wexner score 0), while three patients reported good continence (Wexner score 1–7).

With respect to urinary tract infections, three patients reported complete resolution of postoperative infections. In the remaining patients, infections were generally non-febrile and did not require hospitalisation. QOL, as assessed during follow-up, showed high overall scores, with a mean EQ-5D-5 L index value of 0.93 and a mean VAS score of 76 (SD 16). Patients cited their underlying disease as the primary reason for the reduction in VAS and only secondarily due to CCUD. All patients reported an improvement in their QOL as a result of their post-operative condition.

## Discussion

This study describes the outcomes of converting a SRP into a CCUD via a BSS technique in seven patients, with an average follow-up period of 91 months. The results show that this complex conversion can be carried out safely in a highly specialised centre, leading to stable renal function, fewer severe urinary tract infections, good faecal continence and a satisfactory QOL.

### Safety and functional outcomes of conversion

Conversion from SRP to CCUD was safe, with no high-grade perioperative complications (CDC ≥ 2), despite its technical complexity. Renal function remained stable (0.92 vs. 1.00 mg/dL), indicating no increased risk of deterioration, consistent with previous CCUD studies [17].

A key finding was the reduction in severe urinary tract infections after conversion, with complete resolution in three patients and only non-febrile infections in the remaining patients. This underscores the benefit of conversion in patients with recurrent pyelonephritis (*n* = 5).

Fecal continence was well preserved overall, with four patients achieving a Wexner score of 0 and three demonstrating good continence. This is an important QOL factor for patients considering conversion [18]. QOL was very satisfactory, with a mean EQ-5D-5 L index score of 0.93 and a mean VAS score of 76. All patients reported subjective improvement compared to their preoperative status. These results correspond with those of other studies reporting high satisfaction rates for CCUD [19].

### Critical evaluation of urinary continence

While overall outcomes were favorable, urinary continence remained limited, with only one-third achieving full continence and the remainder reporting mild or moderate incontinence. This should be discussed preoperatively. Reported continence rates for Mitrofanoff channels are higher (89–99%), with revision rates of 18–40% [20, 21]. Importantly, all techniques – appendicovesicostomy (Mitrofanoff), Monti channels, and ileal invagination nipple – are associated with channel-related complications during long-term follow-up, particularly stomal stenosis and the need for secondary surgical revision. Current evidence does not consistently demonstrate a clear superiority of one technique over the others with respect to overall channel durability; rather, the choice between techniques is generally guided by individual anatomical factors, the need for concomitant bowel surgery, and surgeon experience [22]. In the present study, four patients required additional surgical interventions during follow-up, including stomal revision in four patients. This finding is consistent with the literature [17].

These results must be weighed against the alternative of an incontinent urinary diversion, such as an ileal conduit. Although an ileal conduit is technically less complex and associated with lower revision rates, it may substantially impair QOL, particularly in younger patients [23]. Studies show that continent diversions are associated with better physical function and higher patient satisfaction, particularly among younger patients [24]. The avoidance of an external stoma bag and the ability to self-catheterise are perceived by many patients as substantial improvements in QOL [25].

### Indications and surgical complexity

Indications for conversion must be carefully defined. The most common were recurrent pyelonephritis (*n* = 5) and upper tract obstruction with urolithiasis and renal deterioration (*n* = 2), consistent with reported SRP complications [26, 27].

The procedure is highly complex, with operative times > 11 h, and requires an experienced, specialised team. The prolonged operative duration reflects the highly individualised surgical conditions, often in patients with multiple previous abdominal and reconstructive procedures. Key challenges include adhesiolysis, ureteral reimplantation, bowel-sparing resection, and reconstruction of bowel continuity and continence mechanisms. A specialised setting, including urotherapeutic support, is essential for postoperative management.

### Malignancy risk and surveillance

The risk of secondary malignancy at the uretero-intestinal anastomosis is a well-documented long-term complication of SRP and ureterosigmoidostomy. There is an increased risk of colorectal adenocarcinoma of between 8.5 and 42 times the normal rate, with a latency period of 20–38 years [28, 29]. Current guidelines recommend annual endoscopic surveillance beginning no later than 5–10 years after surgery [30]. In the present study, histopathological examination of all resected specimens revealed no evidence of malignancy. However, with a mean follow-up period of 7.5 years, this remains within the typical latency period. It remains unclear whether conversion to CCUD reduces the long-term malignancy risk, as cases of colorectal adenocarcinoma have been reported after re-diversion [31]. This suggests that the carcinogenic process may persist despite surgical conversion [29]. Given this uncertainty, regular endoscopic surveillance appears reasonable, particularly when previously urine-exposed bowel segments are preserved. Therefore, we recommend annual pouchoscopy and regular colonoscopy for all patients following conversion to CCUD, to enable the early detection of neoplastic changes.

### Study limitations

This retrospective exploratory case series includes a small cohort (*n* = 7), reflecting the rarity of the condition and limiting statistical power, yet providing valuable insights to guide clinical practice. In addition, the cohort was heterogeneous with respect to the underlying patient conditions, previous surgical history, and clinical characteristics. Furthermore, some variability in the surgical approach was present, as the procedures were tailored to the individual anatomical and functional requirements of each patient.

Despite the use of validated instruments, QOL assessment remains influenced by the underlying disease, which patients identified as the primary contributor to reduced VAS scores. This highlights the complexity of evaluating outcomes in patients with congenital urological conditions who have undergone multiple prior interventions.

## Conclusion

In summary, this study shows that conversion from SRP to CCUD using a BSS technique is a safe and effective option for carefully selected patients in highly specialised centres. Despite the potential need for revisions to the continence mechanism and the lengthy operative time, this technique offers a valuable alternative to incontinent urinary diversion, particularly for young patients, as it provides a satisfactory QOL and stable renal function. Any possible complications should be clearly communicated to patients preoperatively.

## Data Availability

All data generated or analysed during this study are included in this article. Further enquiries can be directed to the corresponding author.

## Declarations

### AI assistance statement

During the preparation of this manuscript, the authors used ChatGPT (OpenAI, San Francisco, USA; version March 2026) and DeepL (DeepL SE, Cologne, Germany) to improve text clarity and readability. Hand-drawn surgical illustrations were converted into digital figures using AI-assisted tools; all figure content and design were created and verified by the authors. The authors reviewed and edited all content and take full responsibility for the published work.
